# Antinociceptive Synergism of Pomegranate Peel Extract and Acetylsalicylic Acid in an Animal Pain Model

**DOI:** 10.3390/molecules26185434

**Published:** 2021-09-07

**Authors:** José Antonio Guerrero-Solano, Mirandeli Bautista, Claudia Velázquez-González, Minarda De la O-Arciniega, Luis Guillermo González-Olivares, Monserrat Fernández-Moya, Osmar Antonio Jaramillo-Morales

**Affiliations:** 1 Institute of Health Sciences, Academic Area of Pharmacy, Autonomous University of the State of Hidalgo, Circuito Ex Hacienda La Concepción S/N Carretera Pachuca Actopan, San Agustín Tlaxiaca, Hidalgo 42160, Mexico; jose_guerrero@uaeh.edu.mx (J.A.G.-S.); claudiav@uaeh.edu.mx (C.V.-G.); mina@uaeh.edu.mx (M.D.l.O.-A.); 2 Institute of Basic Sciences and Engineering, Academic Area of Chemistry, University of the State of Hidalgo, Carretera Pachuca-Tulancingo km 4.5 s/n, Mineral de la Reforma, Hidalgo 42184, Mexico; lgonzales@uaeh.edu.mx; 3 Life Sciences Division, Nursing and Obstetrics Department, Campus Irapuato-Salamanca, University of Guanajuato, Ex Hacienda el Copal, km. 9 Carretera Irapuato- Silao, A.P. 311, Irapuato, Guanajuato 36500, Mexico; fernandez.m@ugto.mx

**Keywords:** pomegranate peel, *Punica granatum* L., acetylsalicylic acid, combination, antinociceptive, pain, formalin test, isobologram, synergism

## Abstract

Several modern drugs, which are derived from traditional herbal medicine are used in contemporary pharmacotherapy. Currently, the study of drug–plant interactions in pain has increased in recent years, looking for greater efficacy of the drug and reduce side effects. The antinociception induced by intragastric co-administration of the combination of pomegranate peel extract (PoPEx) and acetylsalicylic acid (ASA) was assessed using the isobolographic analysis in formalin test (nociceptive and inflammatory pain). The effective dose that produced 30% of antinociception (ED_30_) was calculated for both drugs from the logarithmic dose–response curves, subsequently generating a curve with the combination on fixed proportions (1:1) of PoPEx and ASA. Through isobolographic analysis, this experimental ED_30_ was compared with the calculated theoretical additive ED_30_. The result was a synergistic interaction, the experimental ED_30_ was significantly smaller (*p* < 0.05) than the theoretical ED_30_. The antinociceptive mechanism of the PoPEx-ASA combination involves the l-Arginine/NO/cGMP pathway, antioxidant capacity, and high content of total phenols. These findings suggest that an interaction between PoPEx and ASA could be a novel treatment for inflammatory and nociceptive pain, also diminish the secondary reactions of ASA.

## 1. Introduction

Pomegranate (*Punica granatum* L.), is a fruit belonging to the Lythraceae family [1]. It is considered one of the oldest fruits for human consumption and is widely grown in tropical and subtropical regions of the world [2,3]. Originally from Iran and its surroundings, the pomegranate has been grown commercially in countries of Europe, Asia, and America [4]. In traditional medicine, every part of pomegranate is used for its therapeutic effects [5,6]. Particularly, pomegranate peel (PoP) has been used for gastrointestinal disorders such as dysentery, diarrhea, stomatitis, ulcers, and bleeding for its astringent properties, it has been used as an antiparasitic agent [7,8,9]. The anti-inflammatory and antinociceptive effects of PoP and pomegranate peel extracts (PoPEx) have been demonstrated in different preclinical studies [10,11,12,13,14,15,16,17,18]. In this context, PoP is a nutrient-rich byproduct that is becoming abundant due to an exponential increase in the production of pomegranate-based products [19]. PoP contains more biological active compounds than edible parts of pomegranate fruit, and it is known that possess phytochemicals with medicinal significance, in particular, a rich variety of phenolic compounds present in it [20,21]. Some of these compounds are fully identified and can be divided into subgroups, such as phenolic acids (caffeic acid, cumaric acid), flavonoids (catequin, gallocatequin, luteolin, quercetin, kaempferol), and tannins (punicalagin, punicalin, granatin A, granatin B, telimagrandin 1, casuarinin, pedunculagin, corilagin, gallagic acid, gallic acid, ellagic acid) among many others [20,22,23]. Many authors attribute to these compounds the anti-inflammatory, antinociceptive and analgesic effects of the PoPEx [13,24,25,26,27,28,29,30,31,32,33]. It is also known that PoPEx reduces gastric ulcerogenic activity and inhibit the progression of the disease [11,14,34,35].

It is widely known that acetylsalicylic acid (ASA) is an orally administered non-steroidal anti-inflammatory agent. ASA binds to and acetylates serine residues in cyclooxygenases, resulting in decreased synthesis of prostaglandin, platelet aggregation, and inflammation. This agent exhibits analgesic, antipyretic, and anticoagulant properties [36]. ASA is the most commonly used analgesic and antipyretic medication worldwide [37]. ASA cause digestive problems such as irritated stomach, upset stomach, stomach ulcers, stomach bleeding, diarrhea, and worsening asthma [38].

On the other hand, analgesics have advantages and disadvantages, none are perfect and none can treat all types of pain, so the effects can be enhanced by effective combinations of agents that act synergistically by one or more mechanisms of action. By activating multiple pain-inhibiting pathways, the combinations can offer potential benefits and provide more effective pain relief, as well as reduce the adverse effects of medications, by using lower doses [39]. In this regard, medicinal plants act as true drugs since the chemical substances that compose them can have biological activity in humans. For this reason, co-administration with drugs can produce variations in the magnitude of their effect. These types of interactions, like those produced between two or more drugs, can be produced by pharmacokinetic or pharmacodynamic mechanisms, so it is of great interest to evaluate the possible interactions when consuming them in combination. Such interactions can result in additive, synergistic, or antagonistic effects [40,41]. Thus, the aim of this study was to investigate the antinociceptive interaction of PoPEx and ASA in the formalin test.

## 2. Results

### 2.1. Time Course of PoPEx and ASA in the Formalin Test

**Figure 1:**
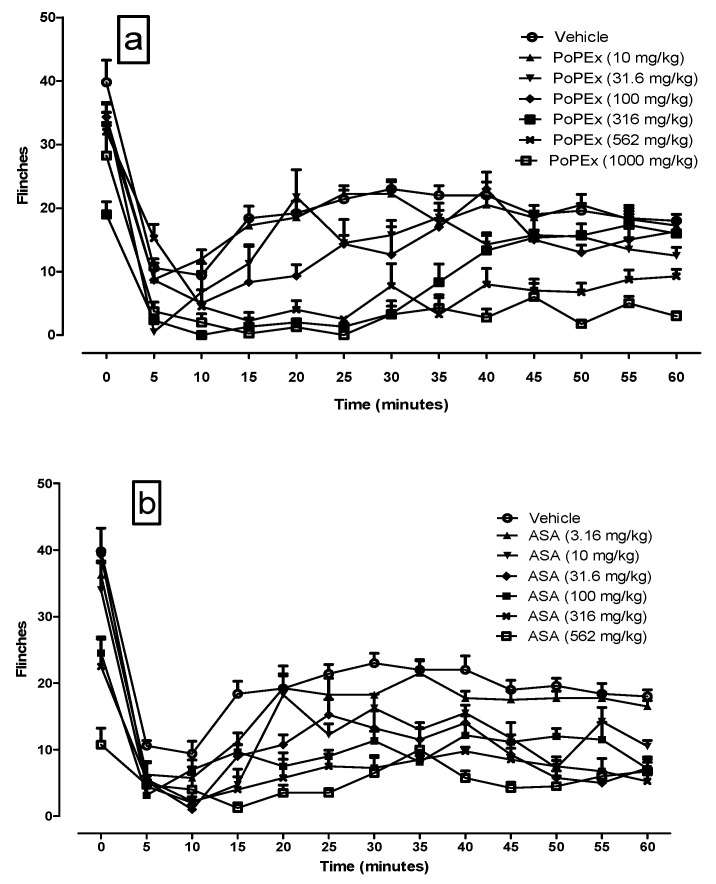
Time course of pomegranate peel extract (**a**) and acetylsalicylic acid (**b**) on the number of formalin-induced paw flinches every 5 min for 1 h. Thirty minutes before the formalin administration, the rats were pretreated with vehicle, ASA, or PoPEx at six different dose in logarithmic increments. The value of *p* ( < 0.05) was not indicated in the figures due to the saturation of lines, however, the statistical differences of the mean ± EE of each measurement time (*n* = 6) in phase 1 were minimal and began to manifest at from the dose of 316 mg for both PoPEx and ASA, and with regard to phase 2, PoPEx began to have a significant effect from 316 mg/kg and ASA from 10 mg/kg weight.

Two percent of formalin administered subcutaneously to the ipsilateral right hind paw of rats produced the expected nociceptive behavior. An immediate nociceptive response (0–15 min; phase 1) was found and after a short quiescent period, an inflammatory response (15 to 60 min; phase 2) that began gradually and continued throughout the entire observation period. The time course showed a typical biphasic response observed in the vehicle group. Figure 1a,b shows the time course of the paw flinches per minute of the different administered logarithmic doses of PoPEx and ASA, compared to the vehicle group. Both, PoPEx and ASA reduced the number of paw flinches in a dose dependent manner.

Subsequently, the area under the curve (AUC) of the time course was analyzed for both phases of the formalin test with the trapezoidal method and from it, the percentage of antinociception was calculated.

### 2.2. Phased and Overall Antinociceptive Effect of PoPEx and ASA in the Formalin Test

Table 1 shows the percentaje of antinociception by phases and overall of PoPEx and ASA in the formalin test, carried out in all the logarithmic dose evaluated in PoPEx and ASA dose–response curve. The dose of all groups of both PoPEx and ASA were found to have a statistically significant (*p* < 0.05) antinociceptive effect in both phases of the formalin test (nociceptive and inflammatory pain) versus vehicle group. However, this effect was higher in phase 2 for both compounds (inflammatory pain).

**Figure 2:**
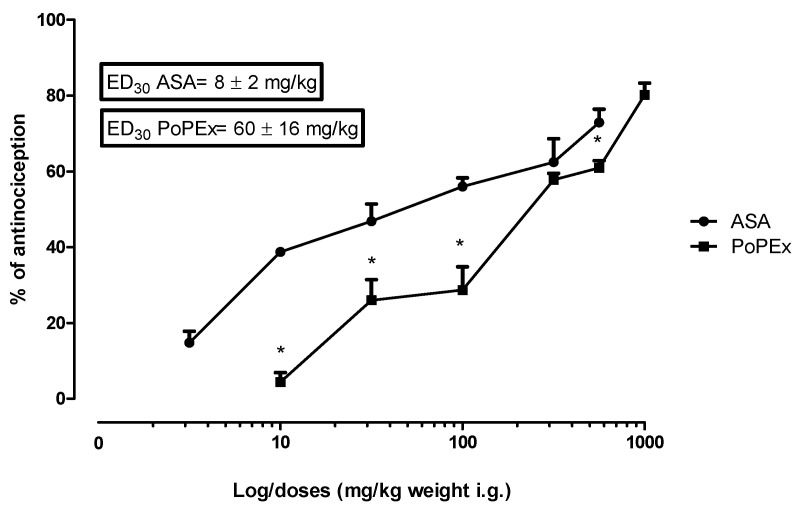
Dose–response curve of PoPEx and ASA. Dose–response curves of the antinociceptive effect of acetylsalicylic acid (ASA) and pomegranate peel extract (PoPEx) from which the ED_30_ was obtained. Each point corresponds to the mean ± EE of 6 animals. * = Statistically significant difference versus ASA groups (*p* < 0.05).

Then the dose response curve for PoPEx and ASA was constructed from the global effect of each dose and drug (Figure 2). By calculating the ED_30_, where the 30% effect is determinated, it was found that ASA has statistically higher potency and efficacy than PoPEx in all points except in the dose of 316 mg/kg (*p* < 0.05). The antinociceptive effect found for both drugs was dose dependend. From these data, the ED_30_ of both drugs was calculated (equeffective doses) and an isobolographic analysis was performed.

### 2.3. Isobologram

The ED_30_ of PoPEx and ASA was calculated from the values obtained from their dose–response curves. The dose response curve of the combination in proportion 1:1 was then performed. The doses used correspond to fractions of the established proportion and are detailed in materials and methods; in this way the theoretical effective dose was formulated. Subsequently, the same calculated doses of the oral combination were experimentally tested in the 2% formalin model. Table 2 shows the percentage of antinociception for each doses. The overall effect found was from 30.4% to 62.8% from a dose dependent manner and a highter percentaje of antinociceptive effect was found in phase 1 than in the drugs evaluated individually.

**Figure 3:**
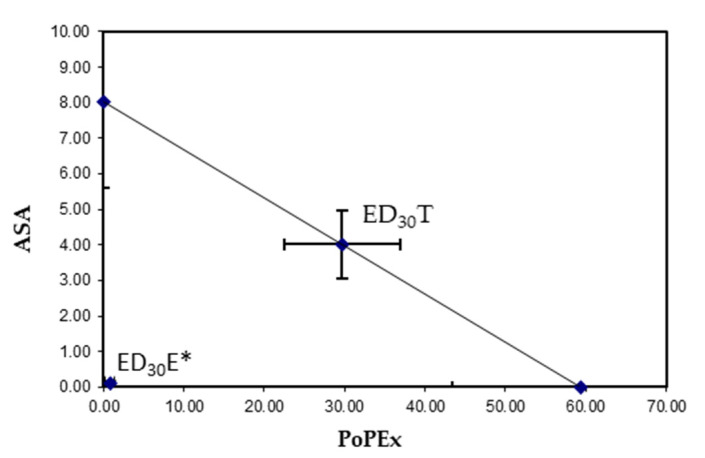
Isobologram of the interaction between acetylsalicylic acid (ASA) and pomegranate peel extract (PoPEx) in the 2% formalin test. The line between the X and Y axes represents the theoretical additive effect that the drug combination would have, the middle point of which is the theoretical additive point of the ED_30_T values of the individual drug. The experimental point (ED_30_E) is observed below the line, close to 0, * = a statistically significant synergistic effect versus ED_30_T (*p* < 0.05).

The isobolographic analysis was performed (Figure 3). In it, the additivity line was drawn between the ED_30_ of the individual drugs and in which the theoretical ED_30_ (ED_30_T) was located in the central part (34 ± 8.11 mg/kg weight). After locating the experimental ED_30_ point (ED_30_E) (0.927 ± 0.63), we found that it was located below the additivity line, with a statistically significant difference (*p* < 0.05) between points, indicating that there are synergistic interaction with the combination. The interaction index (*γ*), which establishes the degree of synergism, was 0.028 ± 0.20; an interaction index less than 1 indicates that there is a potentiation of the effect.

### 2.4. Involvement of the l-Arginine/NO/cGMP Pathway

**Figure 4:**
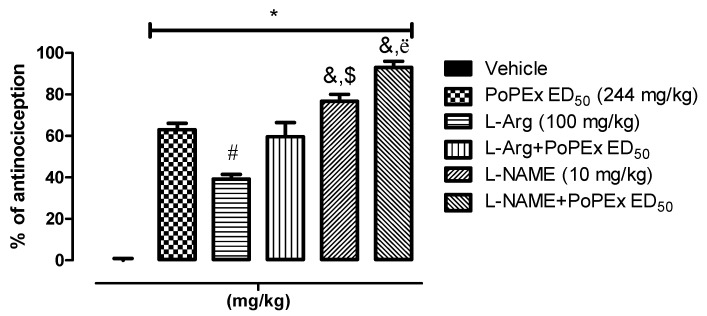
Involvement of the l-Arginine/NO/cGMP pathway. Overall effect of the doses of l-Arginine (100 mg/kg) and l-NAME (10 mg/kg) on the PoPEx ED_50_ was evaluated. The rats were pretreated with an intraperitoneal injection of l-Arginine and l-NAME. * = statistically significant effect (*p* < 0.05) of all groups versus vehicle. # = statistically significant effect (*p* < 0.05) of l-Arginine versus PoPEx ED_50_. &,$ = statistically significant effect (*p* < 0.05) of l-NAME versus PoPEx ED_50_ and l-NAME + PoPEx ED_50_. ë = statistically significant effect (*p* < 0.05) of l-NAME + PoPEx ED_50_ versus l-NAME (determined by one-way ANOVA followed by the Bonferroni test). Each bar corresponds to the mean ± EE of six animals.

To elucidate the mechanism of action of the antinociceptive effect, two experiments were designed with N^ω^-nitro-l-arginine methyl ester (l-NAME), a nitric oxide antagonist. One experiment was designed with PoPEx effective dose 50 (ED_50_) and one with PoPEx + ASA combination 2 (15 mg/kg and 2 mg/kg respectively). The results of the first experiment are shown in Figure 4. In it, it is observed that the central pretreatment with l-Arginine (100 mg/kg i.p.), a precursor to the synthesis of nitric oxide significantly increased (*p* < 0.05) the paw flinches in the formalin test, reducing the percentage of antinociception compared to PoPEx ED_50_. Co-administration of l-Arginine with PoPEx ED_50_ resulted in non-statistically significant difference, compared to the ED_50_ of PoPEx. On the other hand, l-NAME at a dose of 10 mg/kg i.p. had an antinociceptive effect on its own (with a statistically significant difference compared to PoPEx ED_50_) and when co-administered with PoPEx ED_50_ it showed a strong antinociceptive effect with a statistically difference versus PoPEx ED_50_ and l-NAME without PoPEx ED_50_ (*p* < 0.05).

**Figure 5:**
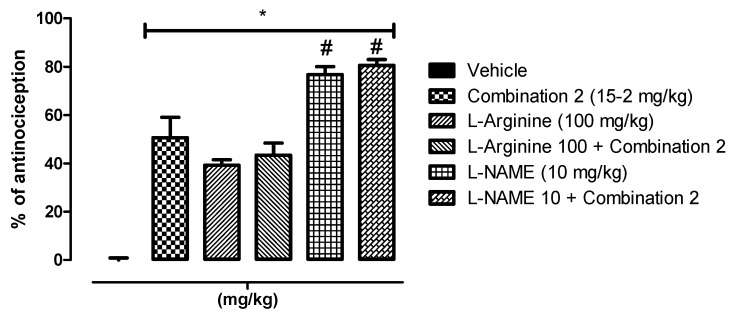
Involvement of the l-Arginine/NO/cGMP pathway. Overall effect of the doses of l-arginine (100 mg/kg) and l-NAME (10 mg/kg) on combination 2 (15 mg PoPEx/2 mg ASA) was evaluated. The rats were pretreated with an intraperitoneal injection of L-arginine and l-NAME. * = statistically significant effect (*p* < 0.05) of all groups versus vehicle. # = statistically significant effect (*p* < 0.05) of l-NAME (10 mg/kg) and L-NAME 10 + Combination 2 versus Combination 2 (Determined by one-way ANOVA followed by the Bonferroni test). Each bar corresponds to the mean ± EE of six animals.

The second experiment was designed in the same way (same groups of l-Arginine and l-NAME) except that PoPEx-ASA combination 2 was used instead of PoPEx ED_50_. The results were consistent with experiment 1 (Figure 5). A statistically significant difference (*p* < 0.05) was found for all groups versus vehicle, and also with l-NAME (10 mg/kg) and l-NAME 10 + Combination 2 versus Combination 2. The results suggest that the antinociceptive effect of both PoPEx alone and the combination with ASA is through the l-arginine/NO/cGMP signaling pathway.

### 2.5. Antioxidant Activity and Total Phenolic Content

Table 3 shows the total phenolic content and antioxidant capacity of PoPEx. In the sample, a free radical scavenging activity was observed in the three tests (DPPH, ABTS, and FRAP), finding activity in PoPEx. Values were expressed as the mean ± SD DPPH and ABTS (µmol TE/g), FRAP (μmol Eq FeSO_4_), and total phenols (mg 100 g^-1^ GAE). Trolox, FeSO_4_, and gallic acid were the reference standards with which the effect of PoPEx was compared and with which the standard curves were elaborated.

## 3. Discussion

In this study, the antinociceptive effect of the combination of PoPEx and ASA was evaluated. To obtain these data, a dose response-curve for each compound was performed first. Once the curve was obtained, the ED_30_ of each compound was calculated to find the ED_30_T and the ED_30_E, with which a curve was once again generated, but this time with PoPEx-ASA in combination, finding a synergistic effect of the compounds administered intra gastric route in the formalin test.

Subcutaneous formalin injection is a well-studied preclinical method for the evaluation of painful chemical stimuli [42]. This provides opportunities to compare the effects of acute and chronic peripheral injury [43]. After injection of 2% formalin in rats, for one hour, the expected biphasic response occurred. Therefore, this test was useful for the evaluation of the proposed treatments, which increased the activity of nociceptors and unleashed a peripheral inflammatory process induced by the injury. In the time course of the test, we found that PoPEx produced a reduction in the number of paw flinches in rats, in a dose–response manner, at all doses administered intragastric route, compared to the vehicle group. This result coincides with that found by Gonzalez-Trujano et al. [44], who evaluated the antinociceptive activity of a whole fruit methanolic extract (10, 30, and 100 mg/kg body weight i.p.) in mice. The time course curve reported by them is quite similar to that found in this study. Similarly, Olapour and Najafzadeh [16], evaluated the antinociceptive effect of a hydroalcoholic extract of pomegranate peel (400 mg/kg body weight i.p.) in the formalin test in mice, and found a statistically significant reduction (*p* < 0.05) of flinches and lick time compared to its control group.

On the other hand, unlike opioids, which have a marked effect in both phases, it is known that non-steroidal anti-inflammatory drugs (NSAIDs) have a greater effect in the second phase of the formalin test [45,46], which explains why ASA had a greater effect in that phase. Although it reduced the number of paw flinches in the first phase (at a rate of 3.4–9.9% antinociception), the second phase exhibited up to 62.5% antinociception. Furthermore, unlike the above studies, in that administered compounds intraperitoneally, we use the intragastric, since it is the most common route of administration for the ASA [47] and because PoPEx is a food by-product, whose use orally for pain is reported in traditional medicine [8,9]. Despite the antinociceptive effect achieved, we think that it would be interesting for future studies to be conducted using other routes of administration in which the effect can be improved.

The antinociceptive effect by phases and overall were calculated from the AUC of the number of paw flinches in relation to the elapsed time in the formalin test, which was expressed as a percentage of antinociception. The results suggest that the analgesic effect of PoPEx is dose dependent, mainly peripheral, and that like ASA, they act poorly through the central pathway and depend on its anti-inflammatory activities. The mechanism of action of ASA is clearly documented [37,47] and our results are consistent with it, however, in the case of PoPEx the mechanisms of action is not entirely clear. There are some studies where the effect of pomegranate was evaluated and especially peel extracts has antinociceptive activity on different types of pain (nociceptive, inflammatory, acute, chronic, or neuropathic) [13,14,15,16,17,18]. The authors indicate that the antinociceptive effect is given by phytochemicals, present in pomegranate; mainly polyphenols and flavonoids (tannins, anthocyanins, and ellagic and gallic acid, among others) but also terpenoids, fatty acids, coumarins, alkaloids, and saponins [48,49].

The main phenolic compounds reported in PoP are ellagitannins (specially punicalagin and gallotanins), flavonoids and free phenolic acids [21,50]. This leads us to think that the effect may be mainly due to this type of molecules. Zeghad et al. [51], reported that an aqueous methanolic extract of pomegranate (1000, 2000, and 3000 mg/kg body weight) could inhibit the production of prostaglandin E2 (PGE2) and nitric oxide (NO) induced by inflammatory cytokines in vivo. This may be caused by inhibition of the release of endogenous nociceptive mediators and the authors suggest that there is a central activity through supraspinal nociceptive activation. In this regard, NO, synthesized from nitric oxide synthase (NOS), is one of multiple modulating components of the nociceptive pathways and mediates pain, both peripheral nociceptive transmission and central sensitization [52]. Gil et al., suggested that antioxidants such as polyphenols and flavonoids optimize the biological effect of NO, stabilizing it and protecting it from free radicals [53]. In addition, ellagitannin metabolites (urolithin A and glucuronide) achieved a decrease in the expression of interleukin-8 (IL-8) and chymosin CCL2, which are inflammatory molecules [26]. Some hydrolyzable tannins (punicalagin, punicalin, among others) have an inhibitory effect on NO production, cyclooxygenase 2 (COX-2) expression and PGE2 [28]. Likewise, punicalagins can modulate the NF-κB signaling pathway [13] and corilagin acts as an antagonist of the transient receptor potential vanilloid 1 (TRPV1) channel [54].

On the other hand, flavonoids present in PoPEx such as quercetin, flavones, resveratrol, kaempferol, and nobiletine inhibit the expression and activity of COX-2 by modulating its transcription [32,55]. Quercetin also suppressed the increased expression of TRPV1 in the spinal cord and dorsal root neurons in a model of neuropathic pain induced by paclitaxel [56]. Finally, free phenolic acids such as ellagic acid and gallic acid, showed an antinociceptive effect in murine models through l-arginine-NO/cGMP/K_ATP_ channels, and also by inhibiting cyclooxygenases, and consequently PGE2. They also decrease the production of IL-6, substance P and bradykinin [25,57,58,59,60].

We thus have that the antinociceptive effect of our extract may be due to its high content of this type of molecules. In this regard, we found in the studies of antioxidant activity and total phenols that we carried out, that PoPEx has a large amount of total phenols (2591 ± 0.06 mg 100 g^−1^ GAE dw). There are reports of total phenolic content of pomegranate peel extracts in which they found 166.83, 152.55, and 85. 48 µg 100 g^−1^ GAE dw [61] and 285–329 µg 100 g^−1^ GAE dw [62] respectively, so we can affirm that our extract has a higher phenolic content than those reported in those studies. In addition, PoPEx has an excellent antioxidant capacity in all three tests (ABTS∙+, DPPH·, and FRAP) taking as reference Trolox and FeSO_4_, which were the ones with which the curves were made. The activity of PoPEx is attributed to his hydrogen donating capability [63]. Our results coincide with Li et al. [64], who reported that the antioxidant activity of the methanolic and ethanolic extracts of pomegranate peel was stronger than the pulp extract in reducing power, therefore they concluded that the peel extract has more potential antioxidant activity. However, despite the existence of previous studies with other extracts of pomegranate peel [20], it is recommended to carry out chromatographic studies of PoPEx, in order to be able to elucidate which groups of molecules are those that make up the entire extract, and thus better clarify the molecular origin of its effect.

The isobolographic analysis demonstrated a strong synergistic interaction between PoPEx and ASA in systemic antinociception, therefore, we are probably facing a synergistic interaction in which a drug has been associated with a group of compounds (PoPEx) with complementary mechanisms of action. In this context, ASA produces an inhibition of COX-2, with the subsequent decrease of prostaglandins. On the other hand, as discussed above in the text, PoPEx has several mechanisms such as inhibiting cyclooxygenases in a non-selective way and therefore the production prostaglandin E2. PoPEx also reduces the expression of pro-inflammatory molecules such as IL-6 and IL-8, reduces the expression of COX-2 and TRPV1, modulates the NF-κB signaling pathway, it is an antagonist of TRPV1 channels and is known to stimulate the l-Arginine/NO/cGMP pathway; however, the whole mechanism of action of this interaction deserves further investigation.

NO is a simple gas formed by a hydrogen atom and an oxygen atom with many physiological roles; in pain, inflammation, immune system, as a vasodilator derived from l-Arginine and an oxygen molecule, which are converted to a NO molecule and a L-citrulline molecule by enzymes called nitric oxide synthases (NOS) [65]. It is known that NO have both an antinociceptive and a pronociceptive effect, depending on the concentration, tissue, or region where its synthesis is stimulated [66] and it has been confirmed by Chen and Levine [67] that NO, has a pronociceptive role in pain states induced by stimuli such as carrageenan, capsaicin, glutamate, formalin, or mechanical stimuli. Lee et al., found that four abundant polyphenols in pomegranate inhibit the expression of NOS [28], so this gives us an idea of the behavior of the extract. We thought that the l-Arginine/NO/cGMP pathway was involved in the PoPEx effect, therefore the experiments were carried out. We found that l-arginine (NOS precursor), generated a moderate percentage of antinociception (around 40%) with significant differences versus vehicle (*p* < 0.05); this result is contradictory to what was cited above (Chen and Levine), since an increase in NO would theoretically generate hyperalgesia. This controversial result can be explained by assuming that l-arginine can reduce [68] or induce [69] hyperalgesia, depending on the l-arginine doses; where low doses increase and high doses inhibit antinociception in the second phase of the formalin test [59]. In this case, one of the lowest doses reported in the literature was used (100 mg/kg), so it can be suggested that the result was due to the dose, the route of administration, the specificity of the drug, or the pharmacokinetics [70]. When L-arginine was co-administered with PoPEx ED_50_ and the PoPEx-ASA combination, there was an increase in the percentage of antinociception, which consolidates the hypothesis that PoPEx may be acting via l-Arginine/NO/cGMP pathway. In this study l-NAME had antinociceptive effect (statistically significant differences versus PoPEx ED_50_ and PoPEx-ASA combination). Reports indicate that l-NAME acts as a partial agonist by acting as a substrate causing rapid induction of inducible nitric oxide synthase (iNOS) gene expression [68,71]. A partial agonist antagonizes NO synthesis in some tissues, but stimulates it in others [68]. The stimulation of NO synthesis may explain the observed antinociception of l-NAME, in models such as those used in this research. Duarte and Ferreira reported that l-NAME caused analgesia in the writhing test and in the carrageenan and PG2 test in mice and rats. They found that N^G^-monomethyl-l-arginine (l-NMMA), another NO synthase inhibitor, significantly blocked the central and peripheral antinociceptive actions of l-NAME, as well as methylene blue (MB), a guanylate cyclase activation inhibitor and potentiated by arginine and by a cGMP phosphodiesterase inhibitor (ODQ) [68]. In our study, when coadministering l-NAME with PoPEx and the PoPEx-ASA combination, an increase in the percentage of nociception was observed, being statistically significant in PoPEx versus all groups and in the combination PoPEx-ASA (except versus l-NAME group).

On the other hand, in this study we did not set out to determine the mechanism of action of the antinociceptive effect observed by ASA on the l-Arginine/NO/cGMP pathway, because it is well known that ASA inhibits COX non-selectively, and this notably attenuates the activity of NOS. Likewise, it has been postulated that this inhibition can serve as a regulatory step of the interaction between the COX and NOS pathways and the changes in Ca^2+^ generating its antinociceptive effect [72].These results suggests a participation via l-Arginine/NO/cGMP pathway. We consider it necessary in the future to delve into this mechanism and use the antagonists l-NMMA and MB to confirm that l-NAME is really inducing the expression of iNOS in this study. Note that the combination group had a similar effect to the ED_50_ of the extract, with a much lower dose.

It should be noted that PoPEx presented mild antinociception (1.6%–9.9%) in phase 1 of the formalin test (Table 1). This effect in phase 1 increased in the combination with ASA and we consider it summative, since it also contributes to the total effect and ASA has been reported to have minimal or no effect in phase 1 [73,74]. We believe that PoPEx has this effect and contributes to the synergistic effect due to the content of alkaloids it presents, such as pseudopelletierine [49,75]. This will have to be tested with a specific antagonist in the future or by isolating the PoPEx alkaloids.

The consumption of PoP and its extracts is considered safe [76,77,78]. In this regard, we consider it necessary to scale the study to a clinical level and evaluate whether the potency and efficacy of the combination, generates the benefits derived from the administration of this combination in the preclinical model.

Finally, it is widely known that most NSAIDs induce gastrointestinal injury [79,80] as secondary reaction; Therefore, a strategy to reduce injuries is to reduce the dose of NSAIDs or use gastroprotective agents at the same time [46]. In this sense, the gastroprotective effect of pomegranate peel is known [11,14,34], so the studied combination complies with both strategies (low doses of ASA and gastroprotection). However, it is strongly suggested that a study of the gastroprotective effect of the combination performed, compared with the administration of ASA in a gastric injury model, be carried out to demonstrate this and generate a genuine interest for the administration of the combination in clinical studies.

## 4. Materials and Methods

### 4.1. Plant Material and Extraction

#### 4.1.1. Collection of the Plant Materials

Pomegranate fruits were collected from the Municipality of Tasquillo, Hidalgo, Mexico (Latitude 20.5485, Longitude: −99.3104 20° 32′5″ North, 99° 18′37″ West. Altitude: 1641), in August 2018. Taxonomic identification was confirmed at the Botany Department in the Institute of Basic Sciences and Engineering and the voucher was deposited at the Pharmacognosy Department.

#### 4.1.2. Preparation of Extracts

The PoP was manually separated and dried in a cool and dry environment for subsequent powdered and maceration in ethanol for three weeks. The methanolic extracts were concentrated under reduced pressure (175 mbar) (Büchi, Switzerland) at bath temperature 35 °C. The final product was weighed and stored in a cool and dry environment until used.

### 4.2. Animals

Experiments were performed on young adult male Wistar rats weighing 180–200 g obtained from the animal facility of the Health Sciences Institute, Autonomous University of the State of Hidalgo. The animals were maintained at temperature and controlled light cycles (12 h dark–light cycle and temperature 27 ± 2 °C), and had free access to food (5008 FormuLab Diet, CA, USA) and drinking water before the experiments. A fasting period of at least 8 h was established before all experiments.

### 4.3. Compound

2% formalin was prepared by diluting a 37% aqueous formaldehyde solution (J.T. Baker, Mexico), in physiological saline. PoPEx was dissolved in 1% Tween 80 (Sigma Aldrich, MA, USA) and was administered intragastric (i.g.). For the dissolution of ASA (Sigma Aldrich, MA, USA), 0.5% carboxymethylcellulose (Sigma Aldrich, MA, USA) was prepared, in which the ASA was suspended and administered i.g. (administration volume for both ASA and PoPEx was 4 mL/kg of weight). Vehicle animals received either saline (4 mL/kg) or 0.5% carboxymethylcellulose (4 mL/kg) i.g. l-NAME (Sigma Aldrich, MA, USA) and l-Arginine (Sigma Aldrich, MA, USA) were prepared by dissolving them into saline solution (PiSA, Mexico) and administered intraperitoneal (i.p.). All solutions were prepared the same day the tests were performed.

### 4.4. Formalin Test

Rats were placed in acrylic cages with a craft paper background to minimize the cold of the worktable for 1 h to acclimate them to their surrounding conditions. A mirror placed behind the acrylic cages allowed for an unobstructed view of the rat’s paws. The antinociceptive activity of the PoPEx was evaluated by the formalin test in rat following the Wheeler-Aceto and Cowan method with modifications [74]. The test consists of measuring for one hour (1 min every 5), the number of flinches of the right hind paw (ipsilateral), after a subcutaneous injection (50 µL of 2% formalin) with a 30 G needle in the dorsal surface of a hind paw, 30 min after the drug administration. The study groups (*n* = 6) were: Group 1: received 1% Tween 80 as a vehicle (Vehicle group). Six groups received PoPEx in doses at logarithmic increments, and six groups received acetylsalicylic acid at doses in logarithmic increments as well (Table 4). The dose of 1000 mg/kg was not evaluated for ASA because it exceeds the LD_50_ reported for this drug [81]. All groups received the same volume of preparations and the route of administration for all was i.g.

#### Measurement of Pain Behavior

The antinociceptive response was measured by evaluating the time course, in which the average number of paw flinches of the different groups were observed, in relation to the vehicle group. Flinch is one of the behaviors related to nociception in the formalin model and is characterized by spontaneous, rapid, and brief shaking or lifting of the leg. Therefore, each episode of tremor, vibration or elevation of the leg was counted as a flinch. Subsequently, the area under the curve of the number of leg flinches in relation to time was calculated [82], and from these results, the percentage of antinociception was calculated, using the formula presented below

(1) $$\%\;Antinociception=\frac{AUC\;vehicle-AUC\;post\;compound}{AUC\;vehicle}\times 100$$

Two types of pain are measured in this test: from 0 to 15 min, it is called phase 1 and represents nociceptive pain, and 15 to 60 min is phase 2, which corresponds to inflammatory pain [43].

### 4.5. Isobolographic Analysis

Isobolographic analysis is a convenient tool to evaluate the interaction between analgesic drugs [83,84]. In the first instance, the effective dose 30 (ED_30_) was calculated for both drugs; ASA and PoPEx, based on their dose–response curves. This dose was considered the theoretical effective dose (ED_30_T) for each drug, generating a theoretical dose–response curve by administering both drugs, simultaneously, in constant proportions (1:1) of ASA (0.5) + PoPEx (0.5). The curves for the response were calculated using the following sequence: (1) ASA ED_30_ + PoPEx ED_30_; (2) (ASA ED_30_ + PoPEx ED_30_)/2; (3) (ASA ED_30_ + PoPEx ED_30_)/4; (4) (ASA ED_30_ + PoPEx ED_30_)/8; (5) (ASA ED_30_ + PoPEx ED_30_)/16; and (6) (ASA ED_30_ + PoPEx ED_30_)/32, as shown in Table 5. The experimental ED_30_ (ED_30_E) was calculated from the dose–response curve of the ASA-PoPEx combination. To determine the interaction type (synergistic, additive, or antagonistic) the value of the ED_30_T was compared with the experimental ED_30_E of the combination and differences were considered (*p* < 0.05). Additionally, the interaction index (*γ*) was calculated as:

(2) $$\gamma =\frac{ED_{30}\;of\;the\;combination\;\left(experimental\right)}{ED_{30}\;of\;the\;combination\;\left(theoretical\right)}$$

Which indicates what portion of the ED_30_ of the individual drug effect accounts for the corresponding ED_30_ in the combination. Values near to 1 correspond to an additive interaction, while values higher than 1 indicate an infra-additive interaction (antagonistic) and values lower than 1 correspond to synergistic interaction (potentiation).

### 4.6. Involvement of the l-Arginine/NO/cGMP Pathway

We determined the role of the nitric oxide/cyclic guanosine monophosphate (NO/cGMP) pathway in the modulation of PoPEx and the PoPEx-ASA combination in antinociceptive activity. The method described by Zakaria et al. [85] was adopted with slight modifications. Two experiments were designed. In experiment 1, five groups (*n* = 6) of rats with the characteristics and conditions described above were formed. The rats of group (1) received PoPEX ED_50_ only; for group (2) the rats were pretreated with 100 mg/kg i.p. of l-arginine 30 min before the formalin test; group (3) received 100 mg/kg i.p. of L-Arginine and 15 min later, PoPEx ED_50_ (244 mg/kg i.g.) was administered, 30 min later the formalin test was performed. Group (4) and (5) were designed in the same way as (2) and (3) but substituting the pretreatment of L-arginine with 100 mg/kg i.p. by l-NAME (10 mg/kg i.p.). Experiment 2 was identical, with the only difference that instead of administering PoPEx ED_50_, the combination PoPEx-ASA (15 and 2 mg/kg respectively) was administered. The results of the formalin tests were analyzed as described above.

### 4.7. Total Phenolic Content and Antioxidant Activity

We weighed 2 mg of the PoPEx sample, transferred it to an amber vial and diluted it in 1 mL of ethanol by gentle shaking.

#### 4.7.1. Total Phenolic Content

The Folin–Ciocalteu method was used to determine total phenols [86]. 100 µL of the PoPEx was mixed with 500 µL of 1:10 diluted Folin–Ciocalteu reagent. Then, 400 µL (7.5%) of sodium carbonate were added and the mixture was incubated for 30 min at room temperature. The absorbance of the mixture was measured at 765 nm in a microplate reader (Biotek Power Wave XS, USA) using gallic acid as a reference standard.

#### 4.7.2. Antioxidant Activity

Antioxidant activity was determined by ABTS∙+, DPPH, and FRAP. The radical cation ABTS∙+ was produced by reacting 7 mmol L^−1^ of ABTS∙+ stock solution with 2.45 mmol L^−1^ potassium persulfate in the dark at room temperature for 16 h before being used. The ABTS·+ solution was diluted with deionized water to an absorbance of 0.70 ± 0.10 at 754 nm. 20 µL of PoPEx sample was added to 980 µL of the diluted ABTS∙+ solution, and absorbance readings were taken after 7 min of incubation at room temperature (754 nm) in the microplate reader [87]. For DPPH an ethanolic solution (7.4 mg 100 mL^−1^) of the stable DPPH∙ radical was prepared and added (500 µL) to 100 µL of PoPEx placed in vials. After the mixture was left to sit at room temperature for 1 h, the absorbance was measured 520 nm in the same microplate reader [88]. FRAP assay was performed according to Benzie and Strain with modifications [89]. A solution was prepared by mixing 25 mL of acetate buffer, 2.5 mL of TPTZ solution and 2.5 mL of FeCl_3_.-H_2_O solution. The mixture was heated to 37 °C before use. PoPEx (30 µL) was mixed with 90 µL of distilled water and 900 µL of the FRAP solution for 10 min in the dark and the absorbance (593 nm) was measured. A standard curve with 5 M ferrous sulfate (FeSO_4_) was used.

### 4.8. Statistical Analysis

The data obtained were expressed as the mean ± SE of the variables. One- and two-way analysis of variance (ANOVA) was carried out, where the difference between the means for each variable was calculated by post-hoc test Bonferroni. The statistical difference between ED_30_E and ED_30_T was determined by Student′s *t*-test. Statistical analysis was performed with Prism 8 for Windows, 2019, GraphPad, San Diego, California. With a significance level of *p* < 0.05.

## 5. Conclusions

These results provide the first evidence that green pomegranate peel extract in combination with ASA produces a potentiation of antinociceptive effects, suggesting its action through the l-Arginine/NO/cGMP pathway, antioxidant capacity, and high content of total phenols. Also, support the possible use of this interaction in the treatment of inflammatory and nociceptive pain. Future studies are necessary to demonstrate other mechanisms of the antinociceptive pathway.

## Figures and Tables

**Table 1:** Phased and overall antinociceptive effect (%) of PoPEx and ASA in the formalin test.

Dose (mg/kg)	Log (D)	Antinociceptive Effect (%)		
		PoPEx Phase 1	PoPEx Phase 2	PoPEx Overall effect
10.00	1.00	1.62	3.23 *	4.83 *
31.62	1.50	6.01 *	19.92 *	25.99 *
100.00	2.00	6.99 *	22.09 *	28.69 *
316.22	2.50	7.22 *	50.23 *	57.81 *
562.34	2.75	7.32 *	55.58 *	60.92 *
1000.00	3.00	7.03 *	72.8 *	80.22 *
		ASA Phase 1	ASA Phase 2	ASA Overall effect
3.16	0.50	3.42 *	10.71 *	14.78 *
10.00	1.00	4.47 *	33.94 *	38.77 *
31.62	1.50	4.99 *	41.73 *	46.85 *
100.00	2.00	6.47 *	49.60 *	56.02 *
316.22	2.50	7.26 *	55.04 *	62.43 *
562.34	2.75	9.94 *	62.55 *	72.94 *

The antinociceptive effect, expressed in percentage, was obtained from the mean of the AUC of the number of paw flinches of each group of animals (*n* = 6) in the 2% formalin test. Drugs were administered intragastrically. * = statistically significant difference (*p* < 0.05) versus the vehicle group.

**Table 2:** Effect of the drug combination (PoPEx/ASA) in the formalin test.

Group	Doses mg/kg (Combination)	Log (D)	Antinociceptive Effect (%)		
			Combination Phase 1	Combination Phase 2	Overall Effect
(1)	34.00	1.53	25.52 *	36.31 *	62.80 *
(2)	17.00	1.23	10.93 *	39.21 *	50.60 *
(3)	8.51	0.93	12.99 *	35.09 *	48.70 *
(4)	4.27	0.62	10.35 *	33.17 *	43.51 *
(5)	2.12	0.32	8.96 *	24.47 *	33.48 *
(6)	1.06	0.02	7.83 *	22.57 *	30.41 *

The antinociceptive effect, expressed in percentage, was obtained from the mean of the AUC of the number of paw flinches of each combination group (*n* = 6) in the 2% formalin test. Drugs were administered intragastrically. * = Statistically significant difference versus vehicle group.

**Table 3:** Antioxidant activity and total phenolic content of PoPEx.

Assay	DPPH·	ABTS·+	FRAP	Total Phenolic Content
Units Standard curve	(µmol TE/g) Trolox (R^2^ = 0.99)	(µmol TE/g) Trolox (R^2^ = 0.99)	(µmol Eq FeSO_4_) FeSO_4_ (R^2^ = 0.99)	(mg 100 g^-1^ GAE) Gallic acid (R^2^ = 0.99)
PoPEx	11805.08 ± 03	11105.72 ± 03	568.82 ± 0.08	2591.01 ± 0.06

The values are means ± SD of three replicates.

**Table 4:** Doses from dose–response curves of PoPEx and ASA before administration of 2% formalin.

Dose (mg/kg Body Weight)			
ASA		PoPEx	
Log (D)	(mg/kg)	Log (D)	(mg/kg)
0.50	3.16	1.00	10.00
1.00	10.00	1.50	31.62
1.50	31.62	2.00	100.00
2.00	100.00	2.50	316.22
2.50	316.00	2.75	562.34
2.75	562.34	3.00	1000.00

**Table 5:** ED_30_ used of the interaction between ASA and PoPEx before administration of 2% formalin.

Dose (mg/kg Body Weight)			
Groups (*n* = 6)	ASA	PoPEx	Total
(1)	4.00	30.00	34.00
(2)	2.00	15.00	17.00
(3)	1.00	7.50	8.51
(4)	0.50	3.75	4.25
(5)	0.25	1.87	2.12
(6)	0.12	0.93	1.06

## Data Availability

To request study data, contact the corresponding author.

## Sample Availability

Samples of the compounds used in this study are available from the authors.
